# Editorial: Microbiome genomics for livestock production

**DOI:** 10.3389/fgene.2022.1000749

**Published:** 2022-09-09

**Authors:** Bruno G. N. Andrade, Marcela M. de Souza, Samat Amat, Luciana C. A. Regitano, Priscila S. N. de Oliveira

**Affiliations:** ^1^ Computer Science Department, Munster Technological University, MTU/ADAPT, Cork, Ireland; ^2^ Embrapa Southeast Livestock, São Carlos, Brazil; ^3^ Department of Microbiological Sciences, North Dakota State University, Fargo, ND, United States; ^4^ Universidade Federal de São Carlos, São Carlos, Brazil

**Keywords:** cattle, genomics, livestock production, microbiome, poultry, porcine

In the Anthropocene era we are living in, the environmental impact caused by the livestock industry must be addressed while meeting the feed standards required by the global population. These goals can be achieved through the improvement of key production phenotypes (i.e., feed efficiency and methane production), and the overall health of individual animals and the herd, reducing the use of natural resources. Such phenotypes, or at least a part of their variance, are the result of interactions between the host and its associated microbiome, and a better understanding of this system can generate a whole new paradigm for the livestock industry.

The microbiota is a community composed of microorganisms (bacteria, archaea, fungi, protozoa, and viruses) that successfully colonise the host’s microenvironments, such as the respiratory, reproductive, and gastrointestinal tracts, being crucial for the host’s overall health and development and their total genomic content is known as the microbiome. The relationship between these microorganisms and the host is crucial for the host’s overall health and development. Such relationship generated a new theoretical framework, themed as the Holobiont theory, in which interactions between the host and its microbiome influence the overall fitness, development, growth and health of the host, shaping its evolution. Considering the importance of uncovering the relationship between livestock species and microbiome to improve animal efficiency, we compiled high-quality papers containing state-of-the-art research in this Research Topic, including novel insights, methodologies, and reviews of the current literature. Research papers provide novel studies of the relationship between specific gastrointestinal bacteria and traits, for instance feed efficiency, methane emission, and the overall health of livestock. The final Research Topic has seven articles covering the aforementioned aspect in bovine, porcine and poultry populations.

The microbiome started to be considered as an important target for animal studies a decade ago and was used to identify key bacteria associated with health and performance and to predict complex phenotypes, such as methane emission and feed efficiency. Ross and Hayes, reviewed and explored the current landscape of this emerging research field, covering data types, methodologies and successful studies in ruminants, pigs, poultry and even humans that were able to predict phenotypes, traits and health status using different microbiome datasets, as well as combining genomic, metabolomic and metagenomic information.

In ruminants, traits such as feed efficiency and methane emission are intimately linked to the microorganisms inhabiting the rumen environment, as they break down complex polysaccharides such as cellulose into short-chain fatty acids (SCFAs), being a target in the quest for therapeutic targets and biomarkers. However, although usually overlooked in multi-gastric animals, the gut microbiome has been described as crucial for homeostasis, immune system training and host health. Andrade et al., identified eight bacterial biomarkers linked with the variation (i.e., increase or decrease) of residual feed intake (RFI) and residual methane emission (RCH_4_) in the gastrointestinal tract of the brazilian Nelore beef cattle using 16s rRNA gene amplicon sequencing data, providing new biomarkers for these traits. Cardinale and Kadarmideen used combinatorial network and machine learning methods to search for metagenomic and host genetic features that could explain variations in the methane emission and RFI in dairy cattle from Holstein and Nordic Red breeds. They identified 26 taxa linked to the variation of the phenotypes, a core of 73 highly heritable microorganisms and a set of SNPs linked to their heritability.

Among factors that can modulate the microbiome composition, feeding strategies, sex and geographical locations are the most significant. Su et al., performed a systematic review and meta-analysis to explore how such factors, including altitude, would impact the microbiome of yaks (*Bos grunniens*). The authors investigated 34 studies and included 982 unique microbiomes in the meta-analysis. They identified significant differences mediated by the described factors, for instance geographic region and diet being the key factors for bacteria from the phyla *Firmicutes* and *Bacteroidetes*. Differences in the gut microbiome provided insights into how they might have helped Yaks to adapt and thrive in high altitude habitats.

The microbiome composition has been consistently linked to the host health, with deviations from a healthy microbiome, also known as dysbiosis, being related to diseases and other health conditions. In fact, the search for immunomodulatory biomarkers linked to the health state has been a central topic for different studies over the years. Alves et al., investigated how the gilts’ vaginal microbiome would change in response to housing systems, such as crates, indoor and outdoor housing, and to an inflammatory challenge caused by intravenously administered lipopolysaccharide (LPS). This study showed that, besides differences in alpha diversity metrics and relative abundances between experimental groups, animals housed in crates presented a reduced microbial diversity, favouring the development of opportunistic bacteria belonging to the *Enterobacter* genus, leading to the development of urogenital infections, and directly impacting the animals’ welfare. In piglets, based on a previous study that also found differences in the microbiome of animals exposed to outdoor soil in early life, De Souza et al., investigated whether this exposure would also modulate the animal immune system. These authors explored the differential expression of specific messenger RNA (mRNA) and microRNA (miRNA) in peripheral blood cells of piglets, resulting from the early exposure to soil microorganisms during the lactation period. A total of 138 mRNAs and 21 miRNAs were identified, and an enrichment analysis revealed terms directly involved with the immune system as affected, such as the connection between T-cell and antigen-presenting cells and the T-cell activation.

The microbiome is composed not only by symbiotic microorganisms with positive effects on the hosts’ health, but also by transient microorganisms and by potential pathogens as well, including different strains of *Escherichia Coli* and other well-known pathogens. Niu et al., investigated the impact of the expression of the Fur gene, which encodes an iron absorption regulatory protein, in the motility and pathogenicity of the Avian pathogenic *E. coli* (APEC), a bacteria responsible for different diseases in birds. This study showed that the Fur protein interacts and binds to the promoter region of the gene flhD, a key flagellum-related gene, affecting the assembly and synthesis of the APEC flagellum. Such molecular mechanisms can offer new options for the prevention and treatment of APEC-related diseases in poultry.

Considering all the above studies, this special edition shows that understanding the role of microbiome in the development of important commercial traits and animal health can have a significant and positive impact in the livestock industry. Additionally, by integrating it as a new variable in animal husbandry or using it as a therapeutic target, we can reduce the environmental footprint while increasing the food yield, thus contributing to the society as a whole. We hope that the reader will find this Research Topic a helpful reference for the state-of-the-art in the emerging field of microbiome genomics in livestock research.

